# Optimization of Physicochemical Properties of Chitosan–Gum Arabic Nanoparticles Containing *Ziziphora clinopodioides* Lam. Extract Using Response Surface Methodology

**DOI:** 10.1002/fsn3.72318

**Published:** 2026-09-04

**Authors:** Anita Ehterami, Hassan Gandomi, Mohammad Kazem Koohi, Negin Noori, Masoud Kazeminia

**Affiliations:** ^1^ Department of Food Hygiene and Quality Control, Faculty of Veterinary Medicine University of Tehran Tehran Iran; ^2^ Division of Toxicology, Department of Comparative Biosciences, Faculty of Veterinary Medicine University of Tehran Tehran Iran

**Keywords:** chitosan, gum arabic, nanoparticles, optimization, response surface methodology, *Ziziphora clinopodioides* L.

## Abstract

*Ziziphora clinopodioides* Lam. extract, as a natural bioactive compound, possesses a wide range of health benefits; however, its inherent instability limits practical applications. To overcome this limitation, nanoparticle‐based delivery systems, particularly chitosan–gum arabic nanoparticles, were employed. The aim of this study was to evaluate the effects of varying concentrations of chitosan (3–9 mg/mL), *Z. clinopodioides* L. extract (3–9 mg/mL), and gum arabic (0.5–1.5 mg/mL), on the physicochemical characteristics of chitosan–gum arabic nanoparticles containing the extract, using a two‐step emulsion–ionic gelation approach. Key parameters, including particle size, zeta potential, and encapsulation efficiency, were assessed by examining both individual and interactive effects. The process was optimized using response surface methodology (RSM) with a Box–Behnken design. The FTIR and XRD analyses were conducted to characterize the nanoparticles. Nanoparticle sizes ranged from 13.90 to 1722.75 nm, while the zeta potentials ranged from 1.63 to 46.67 mV, and the encapsulation efficiencies ranged from 20.51% to 63.66%. Notably, increasing the extract concentration reduced the encapsulation efficiency, while increasing particle size and zeta potential. Higher chitosan concentrations increased nanoparticle zeta potential. In contrast, increasing gum arabic concentration enhanced the encapsulation efficiency. The results demonstrated significant interaction effects among the formulation parameters across all measured responses. Optimal concentrations of chitosan, extract, and gum arabic were determined to be 9, 4.90, and 1.5 mg/mL, respectively, yielding a particle size of 50 nm, a zeta potential of 5.86 mV, and an encapsulation efficiency of 60.63%. The FTIR and XRD analyses confirmed proper encapsulation of the extract. These findings highlight the critical effects of studied parameters on practical characteristics of chitosan–gum arabic nanoparticles loaded with *Z. clinopodioides* L. extract.

## Graphical abstract

This study outlines the preparation of Chs‐GA‐NPs loaded with ZcEx. Dried leaves of the plant were powdered and extracted using an aqueous‐ethanolic method. Chitosan, GA, and Tween‐80 solutions were mixed to form a nanoemulsion, followed by the addition of the plant extract and TPP solution under stirring, which triggered ionic gelation and produced the final NPs. The NPs were then characterized in terms of PS, ZP, and EE, with further structural analysis carried out through FTIR and XRD.

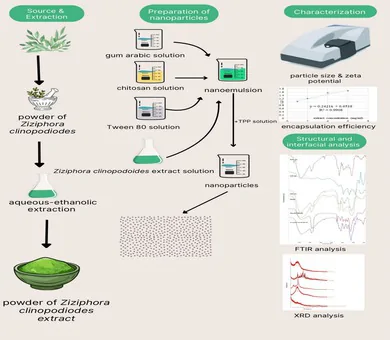

**Abbreviations**
ANOVA: analysis of variance
BBD: Box–Behnken design
Chs: chitosan
Chs‐GA‐NPs: chitosan–gum arabic nanoparticles
Chs‐NPs: chitosan nanoparticles
EE: encapsulation efficiency
Ex: extract
GA: gum arabic
NP: nanoparticle
NPs: nanoparticles
PRESS: Predicted Residual Error Sum of Squares
PS: particle size
RSM: response surface methodology
SD: standard deviation
TPP: sodium tripolyphosphate
Zc: *Ziziphora clinopodioides* L.
ZcEx: *Ziziphora clinopodioides* Lam. extract
ZP: zeta potential

## 1 Introduction

*Ziziphora clinopodioides* Lam. (Zc) is a medicinal plant that belongs to the Lamiaceae family and is widely distributed across various geographical regions including Iran, Anatolia, Armenia, Caucasia, Iraq, Pakistan, Syria, Turkey, Turkmenistan, and West Siberia (Ahmadi et al. 2021). It is a natural product that has long been recognized for its significant benefits to human health and an as effective remedy for various ailments (Ahmeda 2020). Rooted deeply in traditional medicinal practices, this plant has been valued for its antiseptic and anti‐inflammatory agent and also, it serves as an effective digestive aid with carminative, stomach tonic, and appetizer properties (Ahmeda et al. 2020). Beside traditional uses, modern medicine uses this plant for its cardiovascular, anti‐inflammatory, and antimicrobial properties to treat respiratory ailments, gastrointestinal disorders, and infectious diseases. Food industries extensively use Zc extract (ZcEx) as a flavoring agent for foods (Cavalcanti et al. 2022). The antioxidant, antibacterial, and antifungal effects of the extract of Zc have been reported (Hazrati et al. 2020), which pulegone is responsible for such effects. The effective concentration of Zc extract reported by antimicrobial and antioxidant studies varied between 40 and 400 μg/mL (Özkan et al. 2019; Shahbazi 2017). However, many bioactive compounds in Zc are inherently sensitive to environmental factors such as heat, light, and oxygen, leading to rapid degradation (Li et al. 2023). Nowadays, the use of plant extract in preparing green‐synthesized metallic NPs has gained great attention for different applications such as antioxidant, antifungal, antibacterial, anticancer, and antiviral (Ishaq et al. 2020; Yan et al. 2020; Ahmeda et al. 2020; Liu et al. 2020; Dehnoee et al. 2024). Furthermore, nanoencapsulation technology offers significant potential to enhance the beneficial properties of bioactive compounds by increasing their stability and bioavailability (Vozza et al. 2018). Among the various nanoparticle (NP) fabrication methods, ionic gelation between two oppositely charged biopolymers, stands out as an effective approach due to its nontoxic, convenient, and easily controllable properties. Moreover, this method is particularly promising for producing polysaccharide‐based nanocarriers, which play a crucial role in NP formulations (Astutiningsih et al. 2022). In this context, chitosan (Chs) and gum arabic (GA) were chosen as wall materials due to their complementary physicochemical properties (Rajabi et al. 2019). Chs is a natural polymer derived from chitin, which is an effective stabilizing and surface functionalizing biopolymer for NP formulation because of its amino and hydroxyl functional groups (Shi et al. 2021). Due to the ionization of the amine groups of Chs at acidic pH values, it becomes water‐soluble and positively charged. This feature provides a basis for the reaction of Chs with other negatively charged macromolecules such as GA (Astutiningsih et al. 2022). GA is a biocompatible and biodegradable polysaccharide with a highly branched arabinogalactan‐protein structure, which serves as an emulsifying and stabilizing agent in nanoformulations (Rajabi et al. 2019). The physicochemical and functional properties of nanoparticles are fundamentally determined by various factors, particularly the selection and concentration of wall and core materials. Therefore, formulation optimization is essential for achieving nanoparticles (NPs) with desired characteristics. Response Surface Methodology (RSM) is a valuable statistical tool for evaluating the influence of various formulation components on response variables and identifying optimal formulations (Cerrón‐Mercado et al. 2022). Among different RSM designs, the Box–Behnken design (BBD) offers particular advantages in optimization studies by eliminating the need for axial points. This feature simplifies experimentation, reduce costs, and improves efficiency in optimization studies (Astutiningsih et al. 2022). In this study, BBD was employed to optimize the chitosan nanoparticles (Chs‐NPs) formulation loaded with ZcEx by evaluating the effect of different concentrations of Chs, ZcEx, and GA, individually or in interaction, on particle size (PS), zeta potential (ZP), and encapsulation efficiency (EE) to achieve an optimized nanoformulation. Moreover, Fourier‐transform infrared (FTIR) and X‐ray diffraction (XRD) analyses were performed to confirm the encapsulation of the extract.

## 2 Materials and Methods

### 2.1 Material

The aerial parts of Zc were collected from the slopes of the Alborz Mountains, Iran. The plant specimens were taxonomically identified by the Herbarium of the Faculty of Pharmacy, Tehran University of Medical Sciences. The scientific name was verified using the Plant List database (www.theplantlist.org) and was recorded as Herbarium No. 7161‐THE. Chitosan (medium molecular weight, ≥ 75% deacetylation), sodium tripolyphosphate (TPP, technical grade), and Tween 80 were supplied from Sigma‐Aldrich (St. Louis, MO, USA). GA (gum arabic, acacia gum, Mw = 2.95–18.6 × 10^5^ g/mol, moisture = 5.23% dry basis) was supplied from Merck Millipore (Darmstadt, Germany). All other reagents and solvents used in this research were of analytical grade and obtained from Merck (Darmstadt, Germany).

### 2.2 Preparation of the Extract

To prepare ZcEx, an aqueous‐ethanolic solvent (80% ethanol and 20% water, v/v) was used for maximum extraction of bioactive compounds. The air‐dried plant material was ground to a fine powder, and 100 g of powdered Zc was thoroughly mixed with 1 L of the solvent (1:10 w/v ratio) and kept for 24 h at 25°C with gentle mixing. The resulting mixture was filtered through Whatman No. 1 filter paper and subsequently centrifuged at 5000 rpm for 10 min to obtain a clarified solution. The filtrate was concentrated under reduced pressure using a rotary evaporator and dried in an oven at 60°C. The obtained dried Ex was collected in a glass container and stored at 4°C until further use (Vijayakumar et al. 2019).

### 2.3 Experimental Design

The optimization process was conducted using Design‐Expert software (version 11.0.3; Stat‐Ease Inc., Minneapolis, MN, USA) to design the experiments, analyze the relationship between the independent and dependent variables, and optimize the formulation of chitosan–gum arabic nanoparticles (Chs‐GA‐NPs). The RSM method using BBD was employed for 15 experimental runs (R_1_–R_15_), with three coded levels (+1, 0, and −1) for each independent variable (Chs, GA, and ZcEx) and the corresponding dependent variables (PS, ZP, and EE) measured for each run. The experimental levels of the independent variables are summarized in Table 1.

**TABLE 1:** Independent and dependent variables of Box–Behnken design used for nanoencapsulation of *Ziziphora clinopodioides* L. extract by chitosan and gum arabic.

Parameter	Symbol	Levels		
		−1 (low)	0 (medium)	+1 (high)
*Independent variables*				
Chitosan concentration (mg/mL)	*X*_1_	3	6	9
Extract concentration (mg/mL)	*X*_2_	3	6	9
Gum arabic concentration (mg/mL)	*X*_3_	0.5	1	1.5
*Dependent variables*				
Parameter (unit)	Symbol		Objective	
Particle size (nm)	*Y*_1_		Minimize	
Zeta potential (mV)	*Y*_2_		Maximize	
Encapsulation efficiency (%)	*Y*_3_		Maximize	

### 2.4 Preparation of *Z. clinopodioides* L. Extract Loaded Chitosan–Gum Arabic Nanoparticles

The ZcEx‐loaded Chs‐GA‐NPs were synthesized following the ionic gelation method described by Rajabi et al. (2019) with slight modifications. Chs powder was dissolved in 35 mL of 1% (v/v) acetic acid solution with hot plate magnetic stirring for 1 h. Simultaneously, GA was dissolved in distilled water under the same conditions. Then, 5 mL of the GA solution was gradually added to the Chs solution and magnetically stirred for 1 h to obtain a homogeneous mixture. The pH of the resulting mixture was adjusted to 4.7 using 1 N potassium hydroxide (KOH). Subsequently, 1 mL of Tween 80 was added as an emulsifier and stirred for another hour. Then, 5 mL of the ZcEx solution was added, and the resulting suspension was homogenized at 14,000 rpm for 15 min. Finally, 5 mL of a 6 mg/mL (w/v) TPP solution was added dropwise to the homogenized mixture while stirring for 5 min (the final volume for each treatment was set to 50 mL). The NP formation was completed by incubating the suspension on a rotary shaker at 150 rpm at room temperature for 30 min. The NPs were collected by centrifugation at 14,000 rpm for 20 min at 4°C, followed by washing with distilled water to remove unencapsulated materials. The purified NPs were suspended in 50 mL of distilled water and stored at 4°C until further characterization.

### 2.5 Physicochemical Characterization

#### 2.5.1 Particle Size and Zeta Potential

The PS and ZP of the prepared samples were measured by the dynamic light scattering technique with a Zetasizer (HORIBA SZ100, Japan) at a constant temperature of 25°C ± 0.5°C.

#### 2.5.2 Determination of Encapsulation Efficiency

To determine the EE, 1 mL of a 1% (v/v) bile salt solution was added to 1 mL of the NP suspension for each of the 15 treatments in separate test tubes. Each mixture was vortexed for 30 s to ensure thorough dispersion of components. Subsequently, 4 mL of ethanol was added to each tube, the mixture was vortexed again, and centrifuged at 6000 rpm for 10 min. A calibration curve for the Ex concentration was prepared by measuring the UV absorbance of ethanolic solutions with concentrations of 1, 4, 6, 8, and 10 mg/mL over the wavelength range of 200–450 nm. The maximum absorbance (*λ*_max_) was recorded at 305 nm. The absorbance of the supernatant was measured at *λ*_max_ using a UV–Vis spectrophotometer (Shimadzu UV‐1800, Kyoto, Japan) to calculate EE (Kazeminia et al. 2024).

#### 2.5.3 FTIR Analysis

FTIR analysis was performed using a Bruker Tensor 27 (Bruker Optik GmbH, Ettlingen, Germany). The samples were mixed with potassium bromide (KBr) and pressed using a manual pellet press. FTIR spectra were recorded in the wavenumber range of 4000–400 cm^−1^. Spectral interpretation was based on the position and intensity of absorption bands corresponding to different functional groups. Comparison of the spectral patterns of Ex‐loaded samples with blank samples (without Ex) enabled identification of changes resulting from molecular interaction (Rajabi et al. 2019).

#### 2.5.4 XRD Evaluation

XRD analysis was performed using a DX‐2700BH multi‐function diffractometer (Dandong Fangyuan Instrument Co., China). Diffraction patterns were recorded over a 2*θ* angular range of 5°–90° using Cu‐Kα radiation (*λ* = 1.54 Å) at 40 kV and 30 mA (Deepika et al. 2021).

## 3 Results and Discussion

### 3.1 RSM Optimization of *Z. clinopodioides* L. Extract Nanoparticle Preparation

The optimization process was conducted using Design‐Expert software and involved three main stages: data entry and verification, response analysis, and formulation optimization. Table 2 presents the actual values, predicted values, and residuals for 15 experimental runs of the nanoencapsulation process. The actual values were obtained experimentally, the predicted values were predicted by the software model, and residual values were calculated as the difference between the actual and predicted values. The obtained PS, ZP, EE ranged from 13.90 to 1722.75 nm, 1.63 to 46.67 mV and 20.51% to 63.66%, respectively. These findings are comparable to a previous study by Rajabi et al. (2019), who reported PS values between 183 and 295 nm, ZP values between 26.4 and 50.8 mV, and EE values between 29.12% and 52.34% for Chs‐GA‐NPs encapsulating saffron bioactive components.

**TABLE 2:** Actual, predicted, and residual response values from various runs determined by Box–Behnken design for nanoencapsulation of *Ziziphora clinopodioides* L. extract by chitosan and gum arabic.

Std	Run	*X*_1_	*X*_2_	*X*_3_	*Y*_1_			*Y*_2_			*Y*_3_		
					Actual	Predict	Residual	Actual	Predict	Residual	Actual	Predict	Residual
9	1	6	3	0.5	13.90	451.29	−437.39	4.25	5.93	−1.68	40.55	40.20	0.3566
8	2	9	6	1.5	318.67	579.48	−260.81	1.70	−10.12	11.82	63.67	61.04	2.63
1	3	3	3	1	701.73	436.31	265.43	35.25	21.75	13.50	20.51	18.24	2.27
3	4	3	9	1	864.93	1011.14	−146.21	35.00	32.45	2.55	24.35	22.67	1.69
2	5	9	3	1	146.10	32.90	113.20	4.33	6.89	−2.55	53.20	54.88	−1.69
11	6	6	3	1.5	149.33	17.91	131.42	6.37	15.64	−9.27	31.78	32.72	−0.9443
15	7	6	6	1	62.70	455.36	−392.66	7.63	19.41	−11.87	32.36	34.61	−2.25
13	8	6	6	1	604.60	455.36	149.24	3.93	19.41	−15.48	37.31	34.61	2.69
4	9	9	9	1	42.65	341.08	−298.43	1.63	15.13	−13.50	42.44	44.72	−2.27
5	10	3	6	1.5	985.10	829.32	155.78	1.67	13.49	−11.82	26.17	28.80	−2.63
7	11	3	6	1.5	205.10	618.13	−413.03	1.77	6.00	−4.23	22.60	23.93	−1.33
6	12	9	6	1.5	102.50	−205.50	308.00	1.67	−2.56	4.23	51.71	50.38	1.33
12	13	6	9	1.5	1722.75	1179.70	543.05	9.55	7.87	1.68	39.86	40.22	−0.3566
14	14	6	6	1	763.50	455.36	308.14	46.67	19.41	27.26	34.17	34.61	−0.4444
10	15	6	9	1.5	146.77	172.52	−25.76	41.90	32.63	9.27	27.91	26.96	0.9443

Abbreviations: run, run order; Std, standard order; *X*_1_, chitosan (mg/mL); *X*_2_, extract (mg/mL); *X*_3_, gum arabic (mg/mL); *Y*_1_, particle size (nm); *Y*_2_, zeta potential (mV); *Y*_3_: encapsulation efficiency (%).

#### 3.1.1 Analysis of the Effects of Nanoencapsulation Parameters on Different Responses

##### 3.1.1.1 Particle Size

Particle size strongly influences dissolution behavior and the colloidal stability of NPs, making it a key parameter in NP formulation. A reduction in PS leads to enhanced colloidal stability by decreasing the sedimentation rate (Rajabi et al. 2019). Smaller NPs demonstrate superior biofilm penetration, a critical advantage for combating infections (Kazeminia et al. 2024). PS also significantly influences EE and essential oil loading capacity; larger particles typically exhibit reduced essential oil loading, which compromises the release kinetics and therapeutic efficacy of encapsulated bioactive compounds (Pineda and Durango 2018). Conversely, smaller NPs enhance therapeutic outcomes across diverse applications, including cancer treatment, by optimizing essential oil bioavailability and modulating release kinetics, thereby amplifying overall therapeutic efficacy (Astutiningsih et al. 2022). The results of the model fitting for PS under varying concentrations of ZcEx, Chs, and GA are shown in Table 3. Model selection was based on multiple statistical criteria including a lower standard deviation (SD) and a smaller Predicted Residual Error Sum of Squares (PRESS), as well as higher values for the coefficient of determination *R* ^2^, adjusted *R* ^2^, and predicted *R* ^2^ values. *R* ^2^ shows the proportion of data variance explained by the model, adjusted *R* ^2^ corrects this value based on the number of factors to avoid overfitting, and predicted *R* ^2^ indicates the model's ability to predict new data. Accordingly, the selected model for PS was Quadratic.

**TABLE 3:** Results of the model fitness for nanoparticle size analysis by Box–Behnken design used for nanoencapsulation of *Z. clinopodioides* L. extract by chitosan and gum arabic.

Source	Sum of squares	df	Mean square	*F*‐value^b^	*p* ^c^
*Lack of fit tests*					
Linear	1.918E+06	8	2.397E+05	18.99	0.1758
2FI	6.437E+05	5	1.287E+05	10.20	0.2332
Quadratic^a^	71047.65	2	35523.83	2.81	0.3884
Pure error	12624.605	1	12624.605	—	—
*Sequential model sum of squares*					
Mean vs. total	3.509E+06	1	3.509E+06	—	—
Linear vs. mean	9.096E+05	3	3.032E+05	1.41	0.3014
2FI vs. linear	1.274E+06	3	4.246E+05	3.88	0.0742
Quadratic vs. 2FI^a^	5.727E+05	3	1.909E+05	6.84	0.0742
Residual	12624.60	1	12624.60	—	—
Total	6.348E+06	13	4.883E+05	—	—

Source	SD	*R* ^2^	Adjusted *R* ^2^	Predicted *R* ^2^	PRESS
*Model summary statistics*					
Linear	463.10	0.3203	0.0937	−0.5757	4.475E+06
2FI	330.74	0.7689	0.5377	−0.3622	3.868E+06
Quadratic^a^	167.01	0.9705	0.8821	—	—

^a^ Suggested.

^b^ Fisher's function.

^c^ Level of significance.

To further evaluate the selected model for PS, an analysis of variance (ANOVA) was conducted, and the results are presented in Table 4. The Quadratic model for PS was statistically significant (*p* < 0.05), and the lack‐of‐fit test was non‐significant. The *F*‐values of the Quadratic model confirm its statistical significance, with corresponding low *p*‐values indicating a strong effect on the response variables. In this study, the *p*‐values of PS for factors *X*_1_ (Chs), *X*_2_ *X*_3_ (the interaction between Ex and GA), and $X_{1}^{2}$ (square of Chs) were statistically significant.

**TABLE 4:** Analysis of variances of the model selected for nanoparticle size analysis by Box–Behnken design for nanoencapsulation of *Ziziphora clinopodioides* L. extract by chitosan and gum arabic.

Source of variance		Coefficient estimate	Sum of squares	df	Mean square	*F*‐value	*p*
Model	Intercept	684.05	2.756E+06	9	3.062E+05	10.98	0.0369^a^
Linear effect	*X*_1_	−24.36	5.762E+05	1	5.762E+05	20.66	0.0199^a^
Interaction effect	*X*_2_ *X*_3_	850.36	1.446E+06	1	1.446E+06	51.85	0.0055^a^
Quadratic effect	$X_{1}^{2}$	−420.38	4.713E+05	1	4.713E+05	16.90	0.0261^a^
Error effect	Residual	—	83672.26	3	27890.75	—	—
	Lack of fit	—	71047.65	2	35523.83	2.81	0.3884^b^
	Pure error	—	12624.60	1	12624.60	—	—
*R* ^2^		0.9705	SD			167.01	
Adjusted *R* ^2^		0.8821	Mean			519.52	
Predicted *R* ^2^		NA^c^	Coefficient of variation %			32.15	
Adequate precision		11.8496	Final equation in terms of coded factors = 684.05–268.37 * *X*_1_ + 850.36 * *X*_2_ *X*_3_ − 420.38 * *X*_1_^2^				

^a^ Significant.

^b^ Not significant.

^c^ Case with leverage of 1.0000 (predicted *R* ^2^ and PRESS statistic not defined).

**FIGURE 1:**
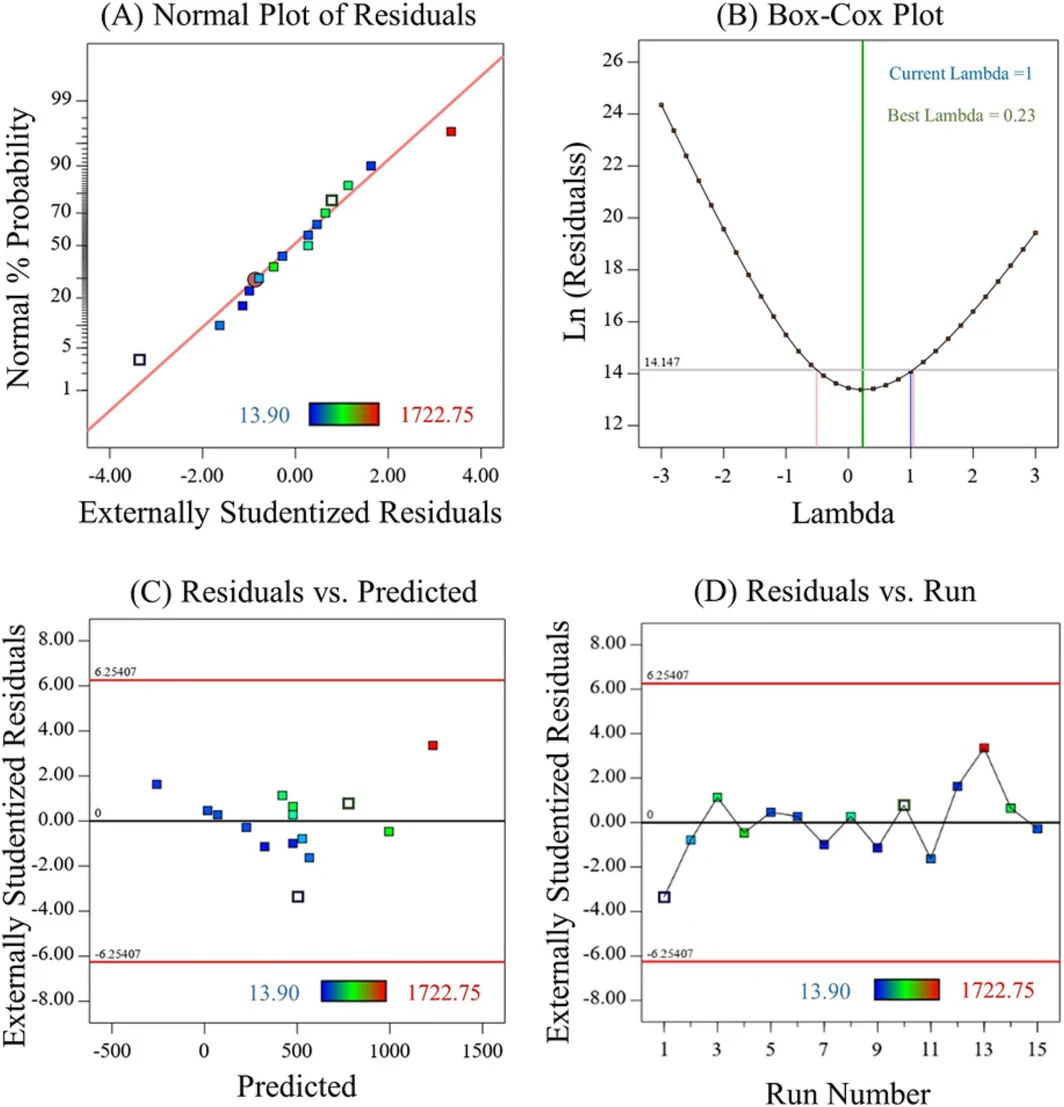
Normal plot (A), Box–Cox plot (B), residuals vs. predicted (C), and residuals vs. run (D) plots for nanoparticle size analysis carried out by Box–Behnken design for nanoencapsulation of *Ziziphora clinopodioides* L. extract by chitosan and gum arabic.

To validate the adequacy of the selected model, diagnostic plots including Normal plot, Box–Cox Plot, residuals vs. predicted and residuals vs. run were assessed and the results are presented in Figure 1. The Normal plot shows that the data points closely follow a straight line, indicating a normal distribution. The Box–Cox Plot (Figure 1B) illustrates that the optimal power transformation (best *λ* = 0.23, indicated by the green line) falls within the confidence intervals (red lines), confirming the model's adequacy. In the residuals vs. predicted (Figure 1C) plot, the residuals are randomly distributed around the zero axis and all data points fall within the specified range (red lines). Furthermore, residuals vs. run (Figure 1D) confirms that all treatments were conducted under consistent experimental conditions, with no systematic errors. Consequently, all these diagnostic plots confirm the adequacy of the Quadratic model for PS response analysis.

**FIGURE 2:**
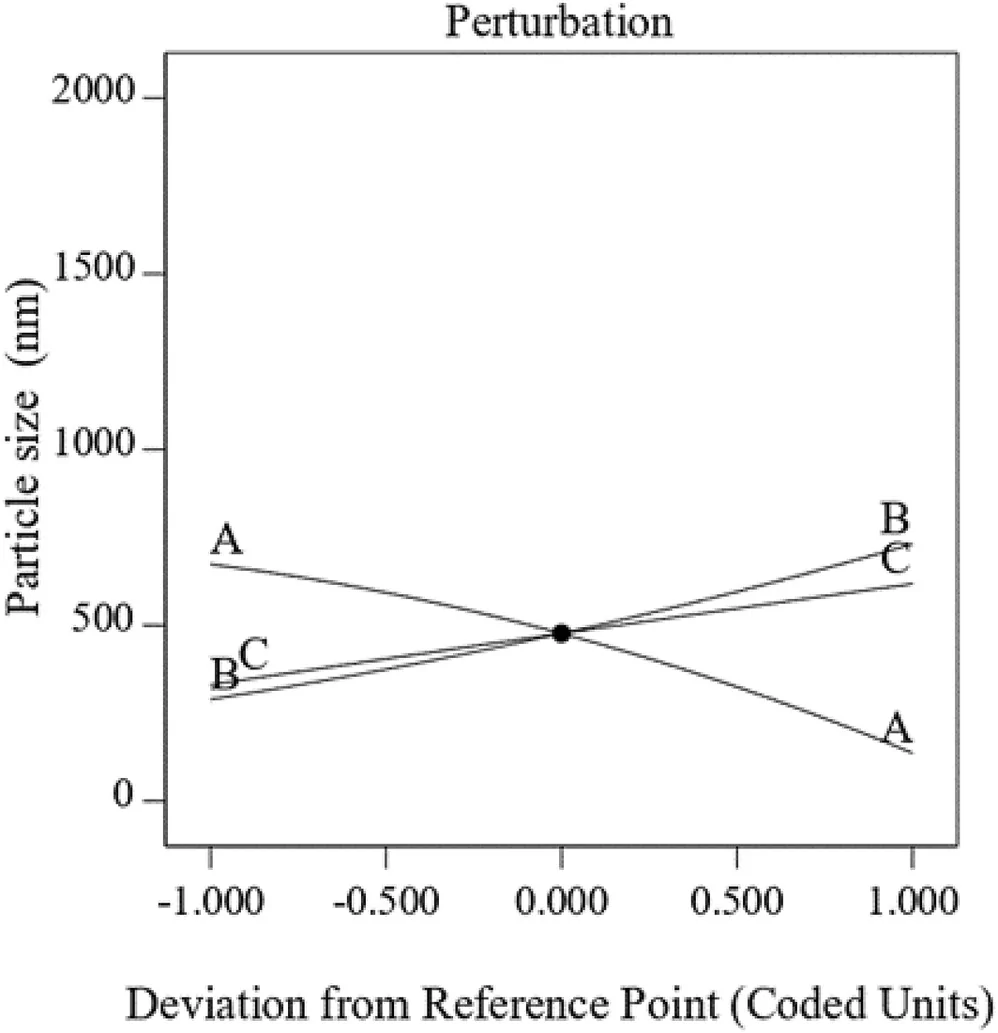
The individual effect of Chitosan (A), extract (B), and gum arabic (C) on particle size of nanoparticle formulations.

Once the experimental model was chosen, the influences of different concentrations of Chs, Ex, and GA on PS, both individually and in interaction, were assessed. In terms of individual effects, as illustrated in Figure 2, Ex exhibited a direct correlation with PS. Similar trends were reported by Hosseini et al. (2013) and Rajabi et al. (2019), who reported that increasing the core material concentration led to insufficient coating, resulting in larger PS. Likewise, Woranuch and Yoksan (2013) in the study about Eugenol‐loaded Chs‐NPs reported an increase in PS. Regarding GA, a direct relationship was observed between concentration and PS, which can be attributed to enhanced electrostatic interactions between the anionic GA and cationic Chs at higher concentrations, leading to increased crosslinking and larger PS (Yousefi et al. 2019).

**FIGURE 3:**
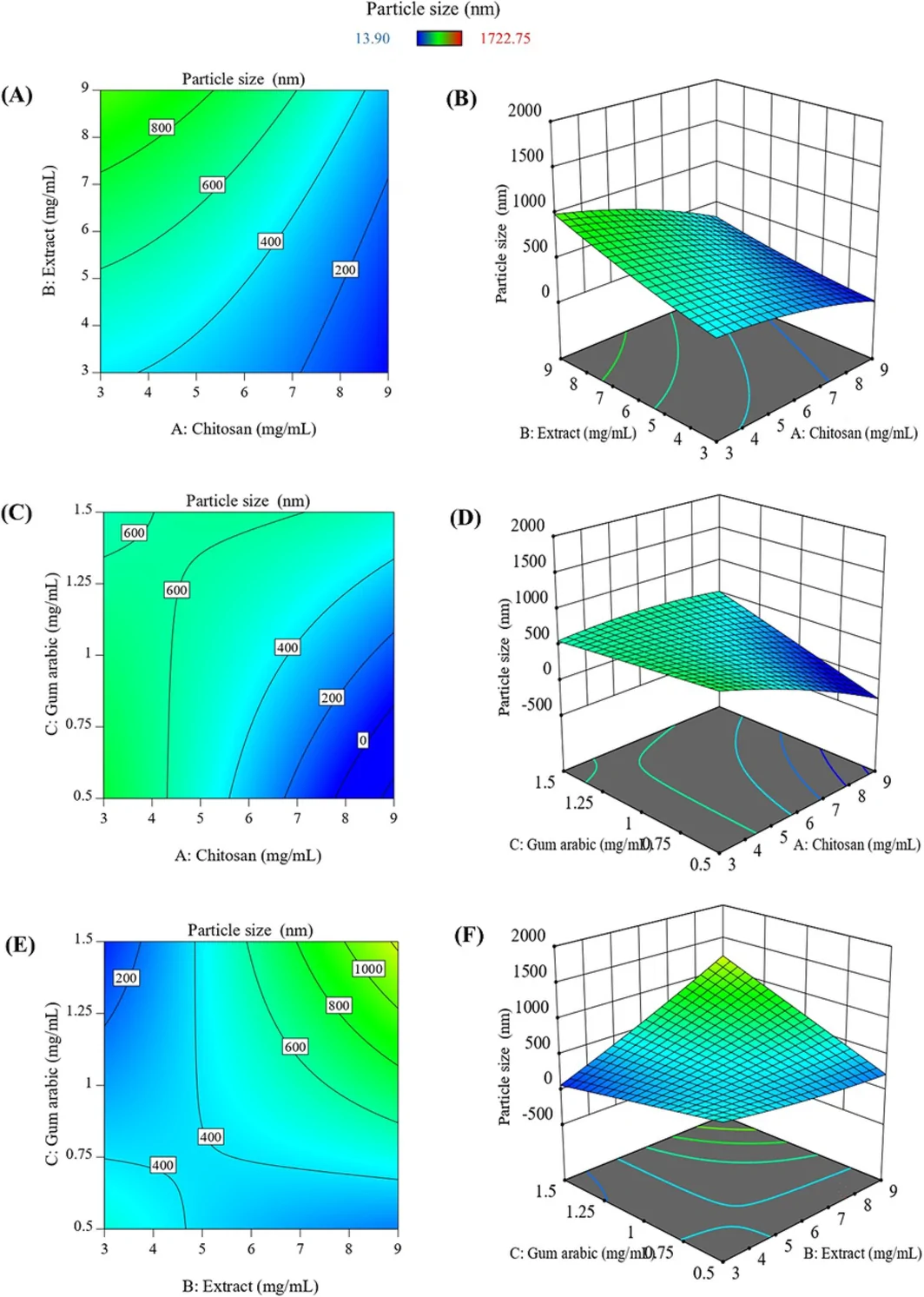
The contour and 3D surface plots for interaction effect of different pairs of independent variables on particle size of nanoparticle formulations.

Figure 3 shows the interactive effects of Chs, Ex, and GA concentrations on PS, presented as three‐dimensional response surface and two‐dimensional contour plots. The interaction effect between Chs and Ex on PS at a medium GA concentration (1 mg/mL) is illustrated through contour diagrams and 3D models (Figure 3A,B). Increasing the Chs to Ex ratio resulted in a decreased PS, while increasing the Ex to Chs ratio led to an increase in PS. The minimum PS was achieved at the highest Chs concentration (9 mg/mL) and the lowest Ex concentration (3 mg/mL). In contrast, maximum PS occurred at the lowest Chs concentration (3 mg/mL) and the highest Ex concentration (9 mg/mL). A similar trend was observed by Hou et al. (2022) in the study about preparation of wide‐domain pH color‐changing nanocapsules. Figure 3C,D show the interaction effect between Chs and GA at a medium Ex concentration (6 mg/mL) on PS. The concentration of GA significantly influenced the effect of Chs on PS. At the lowest GA concentration (0.5 mg/mL), an increase in Chs concentration led to a reduction in PS. However, at higher GA concentrations, this reduction became less pronounced. The smallest PS was achieved at the highest Chs concentration (9 mg/mL) and the lowest GA concentration (0.5 mg/mL), whereas the largest PS was observed at the lowest Chs concentration (3 mg/mL) and the lowest GA concentration (0.5 mg/mL). Similar results were obtained in previous studies, attributing the reduction in PS to strong electrostatic interactions between the negatively charged GA and the positively charged Chs, which form robust ionic bonds that limit particle growth. Conversely, the presence of more polyanions facilitates cross‐linking between amino groups in Chs chains, leading to stronger intermolecular interactions and larger particle formation (Kazeminia et al. 2024). Figure 3E,F illustrate the interaction between Ex and GA at a medium Chs concentration (6 mg/mL) on PS. The PS was significantly influenced by both concentrations of Ex and GA. At low GA concentrations, increasing Ex concentration decreased PS, while at high GA concentrations, increasing Ex concentration increased PS. GA acts as a protective coating, preventing particle aggregation. At high GA concentrations and when interacting with high Ex concentrations, the protective encapsulation became more robust, leading to higher PS values. Therefore, the largest PS was observed at high concentrations of both Ex and GA. The smallest PS was observed at highest GA concentration (1.5 mg/mL) and lowest Ex concentration (9 mg/mL) because GA efficiently encapsulates the Ex, reducing particle growth. In the same way, Jin et al. (2022) in their study on the encapsulation of Zein–GA Complex NPs demonstrated that the PS of complex NP declined when the Zein:GA ratio was decreased from 5:1 to 1:1. They attributed this finding to increased electrostatic contacts and tighter binding in Zein–GA complexes at higher GA concentrations.

##### 3.1.1.2 Zeta Potential

Zeta potential plays a crucial role in assessing the physicochemical stability of particle dispersions, emulsions, and suspensions (Choi et al. 2011). The ZP value quantifies electrostatic repulsion or attraction between particles, governing key colloidal phenomena such as stability, flocculation, and aggregation. Formulations with higher ZP values are desirable, as they enhance electrostatic repulsion and reduce particle aggregation (Honary and Zahir 2013). Particle stability is maintained when the absolute ZP exceeds ±30 mV, whereas values closer to zero increase the risk of flocculation and sedimentation (Kazeminia et al. 2024). The results of the fitting model for ZP under varying concentrations of Ex, Chs, and GA are presented in Table 5. According to the selection parameters mentioned in the previous section, the 2FI model was selected as the most appropriate model for ZP analysis.

**TABLE 5:** Results of the model fitness for nanoparticle zeta potential analysis by Box–Behnken design used for nanoencapsulation of *Ziziphora clinopodioides* L. extract by chitosan and gum arabic.

Source	Sum of squares	df	Mean square	*F*‐value	*p*
*Lack of fit tests*					
Linear	897.57	7	128.22	18.73	0.1761
2FI^a^	154.25	4	38.56	5.63	0.3048
Quadratic	4.11	1	4.11	0.6006	0.5803
Pure error	6.84	1	6.84	—	—
*Sequential model sum of squares*					
Mean vs. total	1194.67	1	1194.67	—	—
Linear vs. mean	1135.30	3	378.43	3.35	0.0763
2FI vs. linear^a^	743.31	3	247.77	7.69	0.0255
Quadratic vs. 2FI	150.14	3	50.05	9.14	0.1003
Residual	6.85	1	6.85	—	—
Total	3234.38	12	269.53	—	—

Source	SD	*R* ^2^	Adjusted *R* ^2^	Predicted *R* ^2^	PRESS
*Model summary statistics*					
Linear	10.63	0.5566	0.3903	−0.1210	2286.56
2FI^a^	5.68	0.9210	0.8262	0.2371	1556.14
Quadratic	2.34	0.9946	0.9705	—	—

^a^ Suggested.

To ensure the robustness of the selected model and evaluate the influence of the tested factors, ANOVA along with lack‐of‐fit tests were conducted. The 2FI model was significant (*p* < 0.05) and the lack‐of‐fit test was non‐significant, which confirmed that the model provided an adequate and reliable fit, as shown in Table 6. Moreover, *p*‐values for *X*_1_ (Chs), *X*_2_ (Ex), *X*_3_ (GA), and *X*_2_ *X*_3_ (interaction between Ex and GA) were all less than 0.05.

**TABLE 6:** Analysis of variances of the model selected for nanoparticle zeta potential analysis by Box–Behnken design for nanoencapsulation of *Ziziphora clinopodioides L.* extract by chitosan and gum arabic.

Source of variance		Coefficient estimate	Sum of squares	df	Mean square	*F*‐value	*p*
Model	Intercept	11.17	1878.61	6	313.10	9.72	0.0123^a^
Linear effect	*X*_1_	−8.02	247.47	1	247.47	7.68	0.0393^a^
	*X*_2_	9.02	475.08	1	475.08	14.74	0.0121^a^
	*X*_3_	−8.02	375.43	1	375.43	11.65	0.0190^a^
Interaction effect	*X*_2_ *X*_3_	−8.62	296.99	1	296.99	9.22	0.0289^a^
Error effect	Residual	—	161.10	5	32.22	—	—
	Lack of fit	—	154.25	4	38.56	5.63	0.3048^b^
	Pure error	—	6.84	1	6.84	—	—
*R* ^2^		0.9210	SD			5.68	
Adjusted *R* ^2^		0.8262	Mean			9.98	
Predicted *R* ^2^		0.2371	Coefficient of variation %			56.89	
Adequate precision		8.2640	Final equation in terms of coded factors = 11.17–8.02 * *X*_1_ + 9.02 * *X*_2_ − 8.02 * *X*_3_ − 8.62 * *X*_2_ *X*_3_				

^a^ Significant.

^b^ Not significant.

**FIGURE 4:**
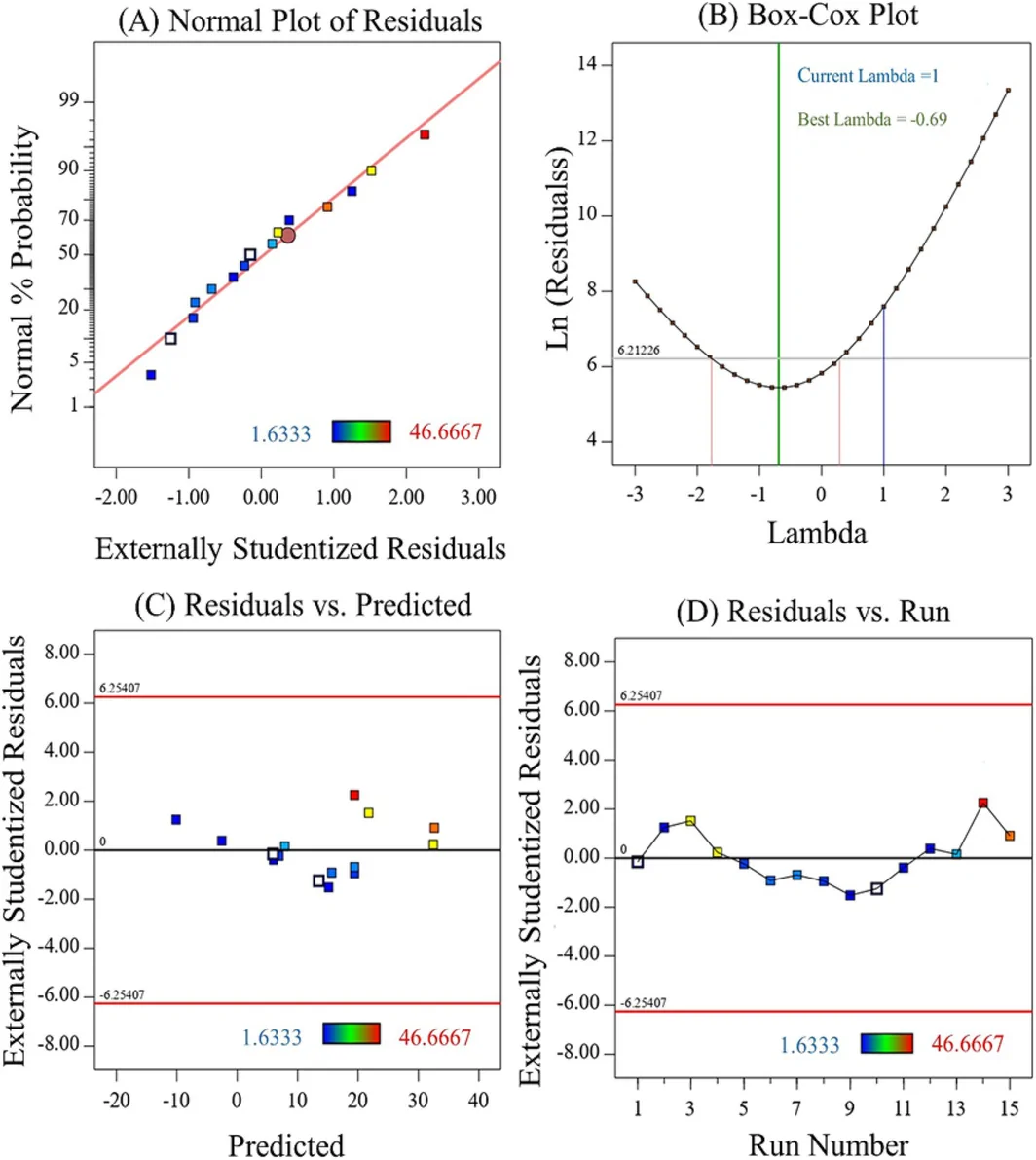
Normal plot (A), Box–Cox plot (B), residuals vs. predicted (C), and residuals vs. run (D) plots for nanoparticle zeta potential analysis carried out by Box–Behnken design for nanoencapsulation of *Ziziphora clinopodioides* L. extract by chitosan and gum arabic.

Additional evaluations of the model's adequacy were conducted through diagnostic plots, including the Normal plot, Box–Cox Plot, residuals vs. predicted, and residuals vs. run, as depicted in Figure 4. In the Normal plot (Figure 4A), data points closely aligned along a straight line, indicating normally distributed residuals with minimal deviations. The Box–Cox Plot (Figure 4B) demonstrates the power transformation, with the optimal lambda value (−0.69, represented by the green line) falling within the confidence interval (red lines), further validating the model's adequacy. In the residuals vs. predicted plot (Figure 4C), the residuals are randomly and symmetrically distributed around the zero axis and remain within the specified confidence limits, confirming the absence of heteroscedasticity or systematic bias. Finally, the residuals vs. run plot (Figure 4D) confirms consistent treatment conditions without patterns or drifts, reinforcing the reliability of the 2FI model for ZP response analysis.

**FIGURE 5:**
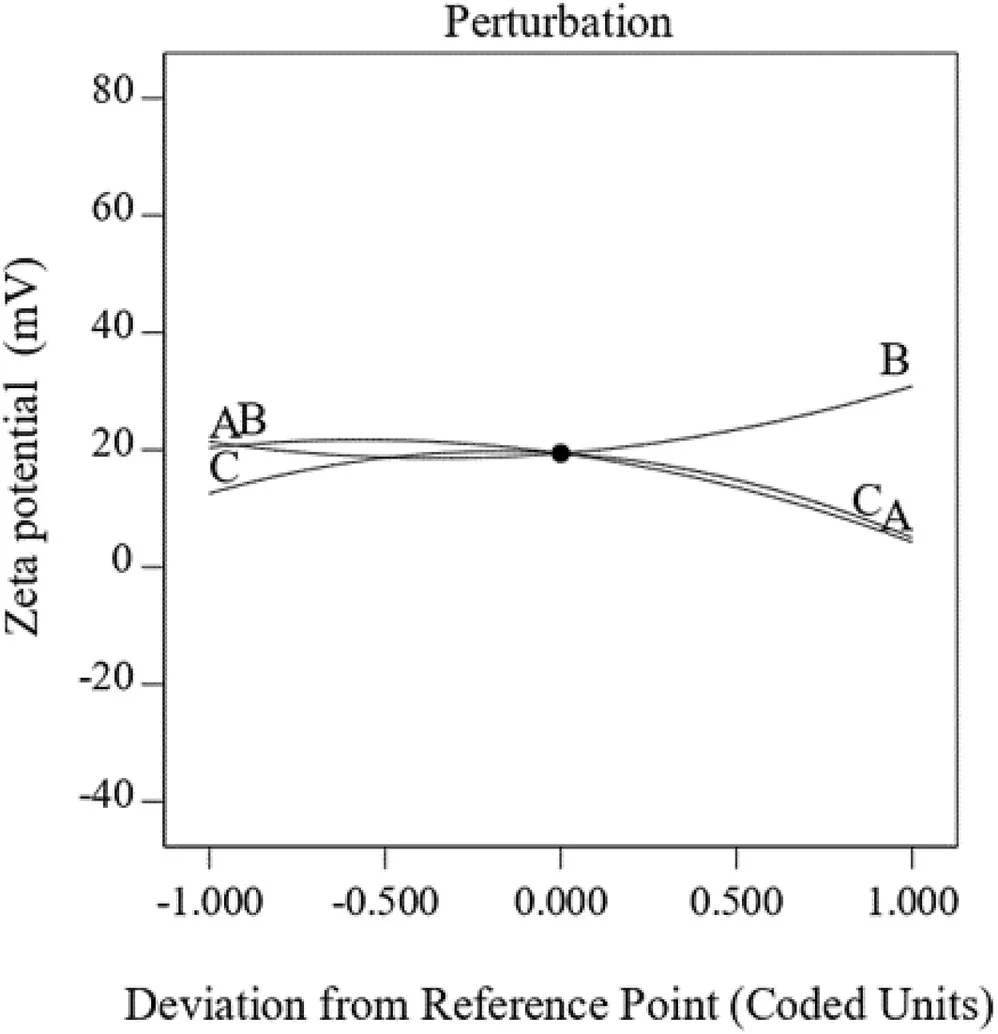
The individual effect of chitosan (A), extract (B), and gum arabic (C) on zeta potential of nanoparticle formulations (the concentration of chitosan, extract, and gum arabic = 6 mg/mL).

After the model was chosen, the effects of experimental factors (Chs, Ex, and GA) on ZP were examined. The individual effects of each experimental factor at different concentrations on ZP are illustrated in Figure 5. Overall, an increase in Chs concentration led to a decrease in ZP, a trend that was more pronounced at medium to high levels. This trend aligns with the findings of Gan et al. (2005), who observed decreased ZP with increasing Chs concentration in Chs‐NPs designed for gene transfer. Conversely, an increase in Ex concentration raised ZP, particularly at medium to high levels. This can be attributed to the presence of a capping layer of phytoconstituents in Ex, which results in a similar surface charge, prevents aggregation, and increases stability (Kazeminia et al. 2024). This is in agreement with the findings of López‐Meneses et al. (2018) about *Schinus molle* L. essential oil‐loaded Chs‐NPs. Furthermore, increasing GA concentration from low to medium levels slightly increased ZP. Chawla et al. (2020) in the study about GA capped copper NPs reported a similar trend in GA‐capped copper NPs, attributing enhanced electrostatic stabilization to arabinogalactan proteins binding with polysaccharide moieties extending into the aqueous solution. However, increasing the GA concentration from medium to high levels altered the electrostatic balance, thereby causing a decrease in ZP. Similarly, Astutiningsih et al. (2022) in the study about optimization of saffron essential oil NPs reported that higher GA concentrations contribute to a decline in ZP during the optimization of saffron essential oil using Chs‐GA‐NPs.

**FIGURE 6:**
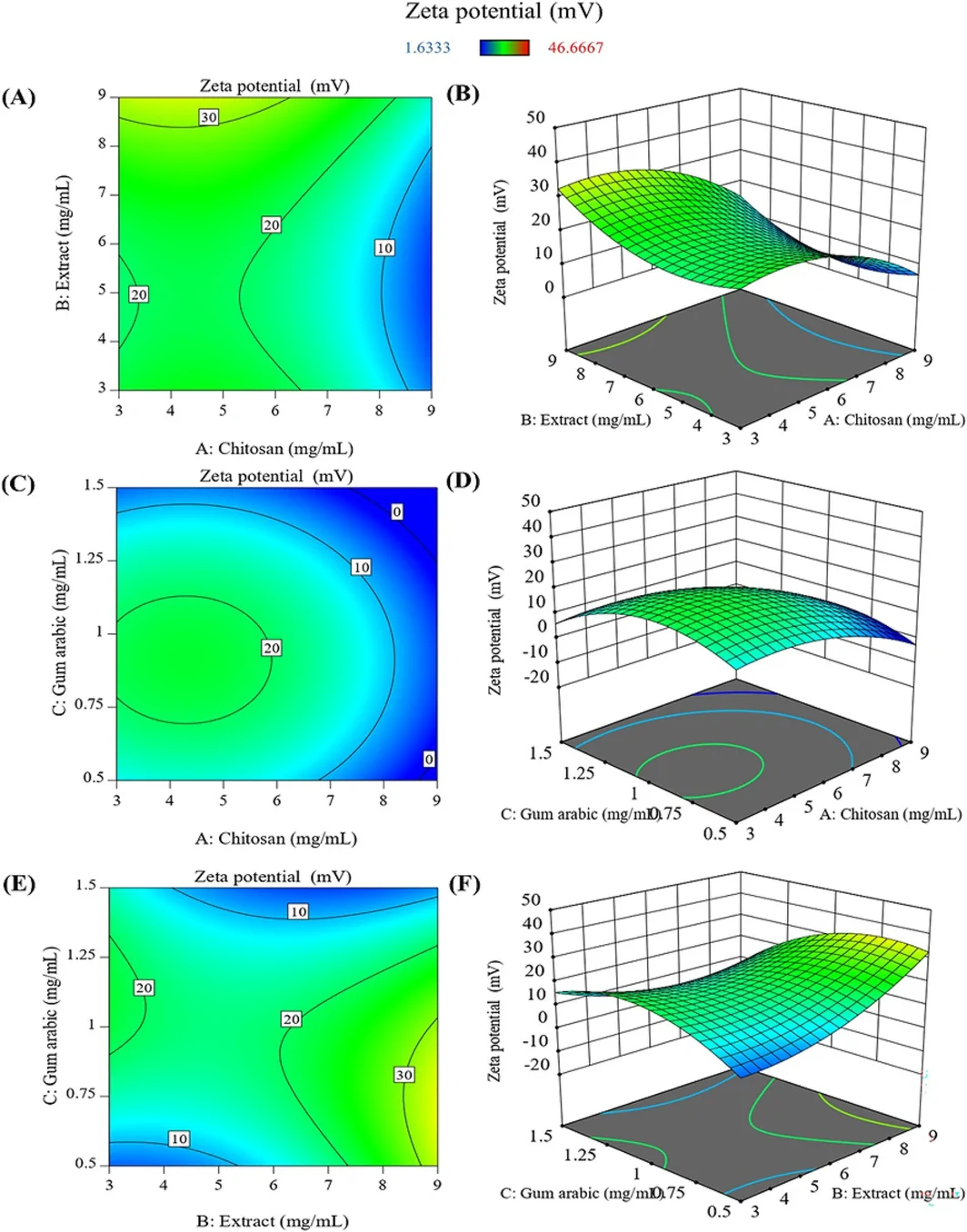
The contour and 3D surface plots for the interaction effect between different pairs of independent variables on the zeta potential of nanoparticle formulations.

Figure 6 illustrates how the Chs, Ex, and GA concentrations affected the ZP of the NPs, shown through 3D response surface and contour plots. The interaction effect between Chs and Ex at medium GA concentration (1 mg/mL) is presented as contour and 3D plots in Figure 6A,B. In general, as the Chs concentration increases, ZP decreases, presumably due to enhanced electrostatic neutralization between Chs and Ex. The minimum ZP was observed at the highest Chs concentration (9 mg/mL) and medium Ex concentrations, while the highest ZP occurred at low‐to‐medium Chs concentrations and the presence of the maximum Ex concentration (9 mg/mL). This observation aligns with the findings of Batista et al. (2019) in the study about characterization of Chs microparticles of bioactive peptides. The two‐dimensional and three‐dimensional models of the GA and Chs interaction effect at a medium concentration of Ex (6 mg/mL) on ZP are shown in Figure 6C,D. The lowest ZP was observed at high Chs concentrations regardless of GA concentration, as well as at high GA concentrations regardless of Chs concentration. The highest ZP was found at medium to low concentrations of Chs and GA and it decreased radially as their concentrations moved away from the central region. These results are congruent with Lobato‐Guarnido et al. (2023) in the study about synthesis and characterization of Chs‐GA‐NPs for encapsulation of oregano essential oil. The interaction effect between Ex and GA at a medium concentration of Chs (6 mg/mL) on ZP is shown in Figure 6E,F. The highest ZP was observed at high Ex concentrations and medium to low GA concentrations. The lowest ZP values were recorded under two conditions: low concentrations of both GA (0.5 mg/mL) and Ex (3 mg/mL), and high GA concentration (1.5 mg/mL) in the interaction with medium Ex concentration (6 mg/mL). These findings align with those reported by Astutiningsih et al. (2022), who demonstrated that increasing the GA‐to‐core material ratio in saffron essential oil‐loaded Chs‐GA‐NPs led to decreased ZP values.

##### 3.1.1.3 Encapsulation Efficiency

EE represents the percentage of core material successfully entrapped within the NPs relative to the total amount of core material used in the formulation (Kazeminia et al. 2024). EE is largely governed by multiple factors, including the concentrations of wall materials and their interactions with the core material (Kurek et al. 2024).

This study examined the impact of varying concentrations of Chs, Ex, and GA on EE. The results of the statistical model for EE are presented in Table 7. Based on the aforementioned model selection criteria, the 2FI model provided the best model for EE.

**TABLE 7:** Results of the model fitness for nanoparticle encapsulation efficiency analysis by Box–Behnken design used for nanoencapsulation of *Ziziphora clinopodioides* L. extract by chitosan and gum arabic.

Source	Sum of squares	df	Mean square	*F*‐value	*p*
*Lack of fit tests*					
Linear	0.0387	9	0.0043	4.43	0.1976
2FI^a^	0.0084	6	0.0014	1.44	0.4653
Quadratic	0.0013	3	0.0004	0.4430	0.7478
Pure error	0.0019	2	0.0010	—	—
*Sequential model sum of squares*					
Mean vs. total	16.97	1	16.97	—	—
Linear vs. mean	0.2469	3	0.0823	22.26	< 0.0001
2FI vs. linear^a^	0.0304	3	0.0101	7.85	0.0091
Quadratic vs. 2FI	0.0071	3	0.0024	3.65	0.0987
Residual	0.0019	2	0.0010	—	—
Total	17.26	15	1.15	—	—

Source	SD	*R* ^2^	Adjusted *R* ^2^	Predicted *R* ^2^	PRESS
*Model summary statistics*					
Linear	0.0608	0.8585	0.8200	0.7038	0.0852
2FI^a^	0.0359	0.9641	0.9372	0.8375	0.0467
Quadratic	0.0254	0.9888	0.9685	0.9130	0.0250

^a^ Suggested.

To comprehensively assess the adequacy and predictive performance of the selected model, an ANOVA and lack‐of‐fit tests were conducted, and corresponding results are presented in Table 8. The ANOVA results revealed that the Chs (*X*_1_), the interaction between Chs and Ex (*X*_1_ *X*_2_), and the interaction between Ex and GA (*X*_2_ *X*_3_) were found to be significant (*p* < 0.05). The *p*‐value for the lack‐of‐fit test was greater than 0.05, indicating no significant lack of fit and confirming the appropriateness of the 2FI model.

**TABLE 8:** Analysis of variances of the model selected for nanoparticle encapsulation efficiency analysis by Box–Behnken design for nanoencapsulation of *Ziziphora clinopodioides* L. extract by chitosan and gum arabic.

Source of variance		Coefficient estimate	Sum of squares	df	Mean square	*F*‐value	*p*
Model	Intercept	1.06	0.2773	6	0.0462	35.85	< 0.0001^a^
Linear effect	*X*_1_	0.1751	0.2453	1	0.2453	190.23	< 0.0001^a^
Interaction effect	*X*_1_ *X*_2_	−0.0432	0.0074	1	0.0074	5.78	0.0429^a^
	*X*_2_ *X*_3_	0.0652	0.0170	1	0.0170	13.18	0.0067^a^
Error effect	Residual	—	0.0103	8	0.0013	—	—
	Lack of fit	—	0.0084	6	0.0014	1.44	0.4653^b^
	Pure error	—	0.0019	2	0.0010	—	—
*R* ^2^		0.9641	SD			0.0359	
Adjusted *R* ^2^		0.9372	Mean			1.06	
Predicted *R* ^2^		0.8375	Coefficient of variation %			3.38	
Adequate precision		17.7950	Final equation in terms of coded factors = 1.06 + 0.1751 * *X*_1_ − 0.0432 * *X*_1_ *X*_2_ + 0.0652 * *X*_2_ *X*_3_				

^a^ Significant.

^b^ Not significant.

**FIGURE 7:**
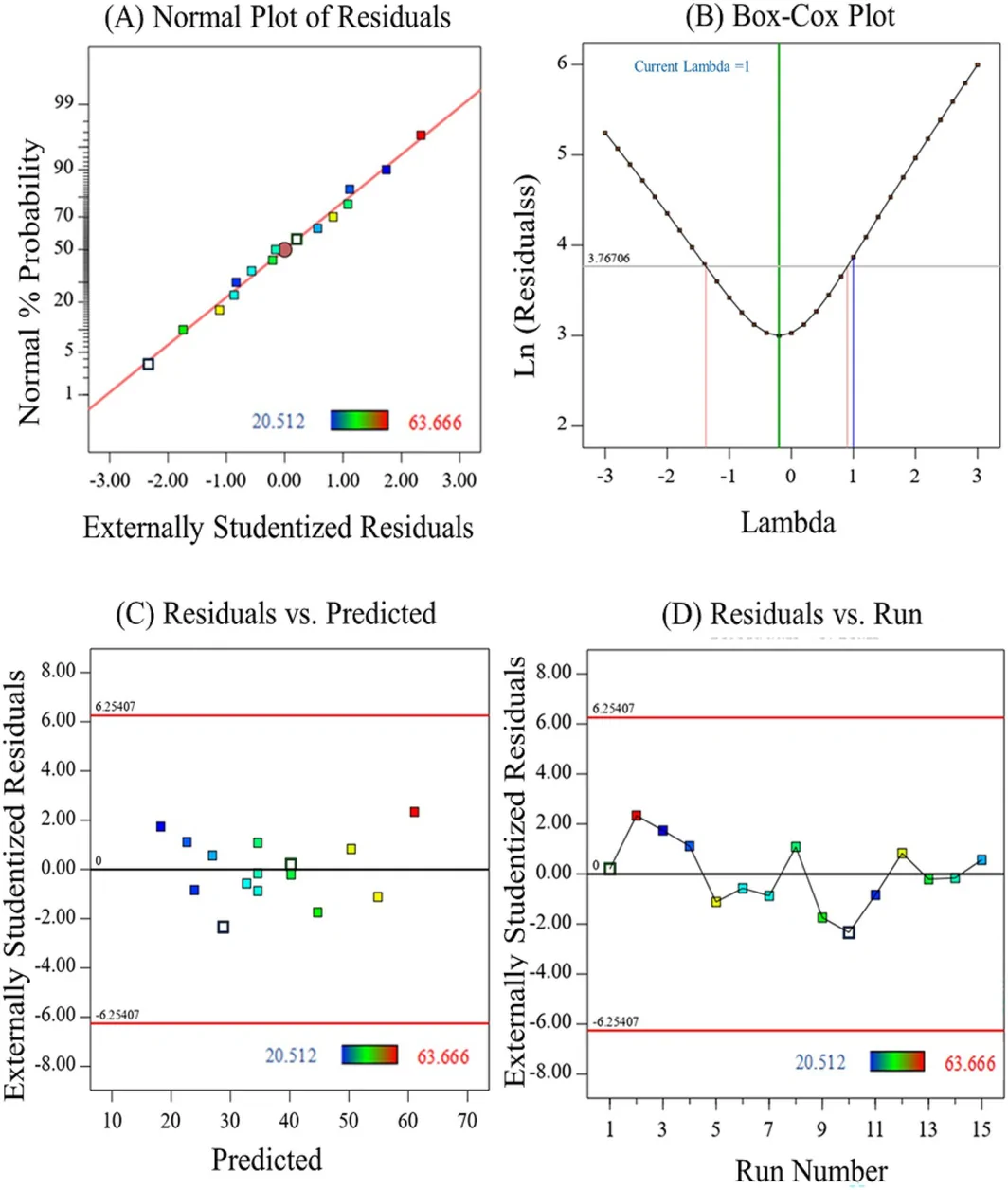
Normal plot (A), Box–Cox plot (B), residuals vs. predicted (C), and residuals vs. run (D) plots for nanoparticle encapsulation efficiency analysis carried out by Box–Behnken design for nanoencapsulation of *Ziziphora clinopodioides* L. extract by chitosan and gum arabic.

The Normal plot (A), Box–Cox Plot (B), residuals vs. predicted (C), and residuals vs. run (D) are shown in Figure 7, validated the adequacy of the 2FI model for EE by showing normally distributed residuals, optimal Box–Cox transformation (*λ* = −0.24), random residual distribution without heteroscedasticity, and consistent experimental conditions throughout the study.

**FIGURE 8:**
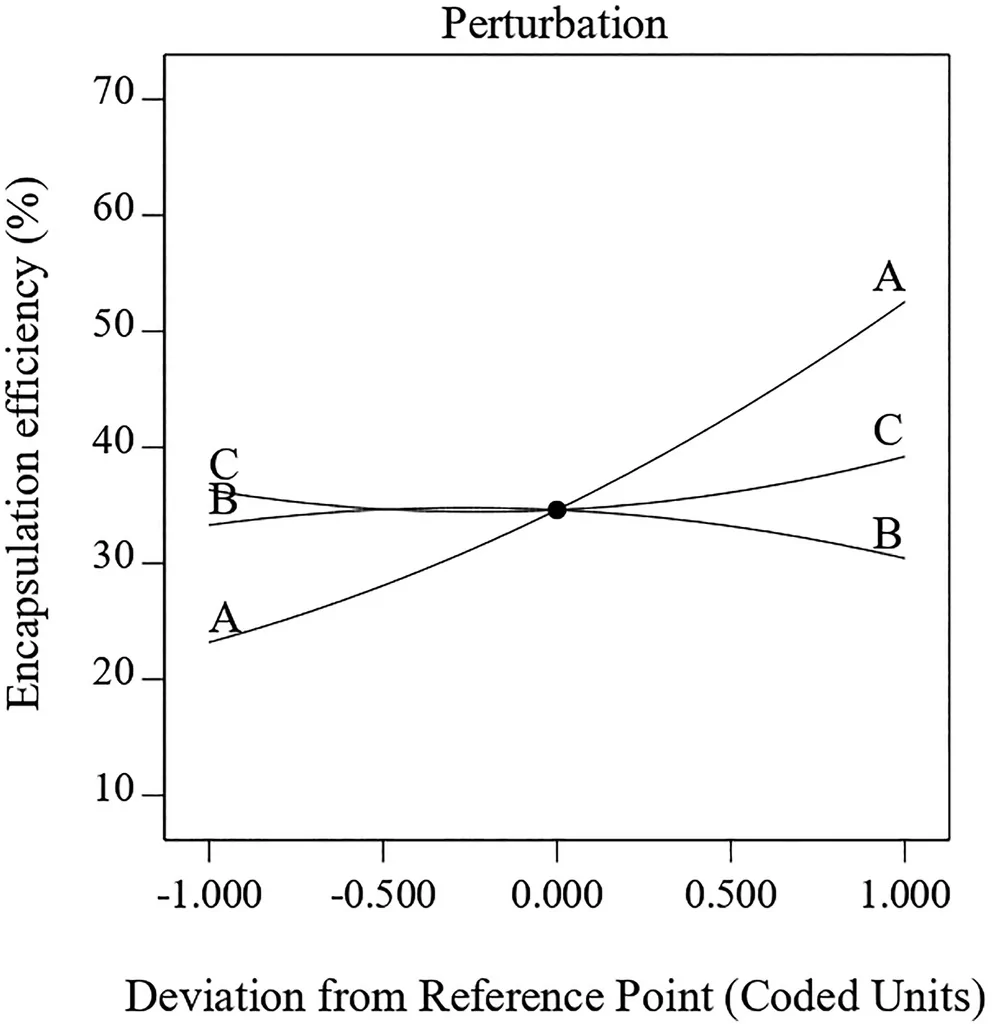
The individual effect of chitosan (A), extract (B), and gum arabic (C) on encapsulation efficiency of nanoparticle formulations (the concentration of chitosan, extract, and gum arabic = 6 mg/mL).

Following model validation, the individual and interactive effects of Chs, Ex, and GA on EE were systematically evaluated, as illustrated in Figure 8A. A direct positive correlation was observed between increasing Chs and GA concentrations and EE. Alam et al. (2023) reported a similar positive correlation between Chs concentration and EE in *Sida cordifolia* ‐loaded Chs‐NPs. Similarly, Hudiyanti et al. (2022) demonstrated a direct relationship between GA concentration and EE in the study about in vitro evaluation of curcumin encapsulation in gum arabic dispersions.

Ex concentration showed a lower effect on EE than Chs; however, optimal EE was achieved at intermediate Ex concentrations, with efficiency declining at both lower and higher concentrations. These results are consistent with findings reported by Kurek et al. (2024) in the study about encapsulation efficiency of food bioactive ingredients.

**FIGURE 9:**
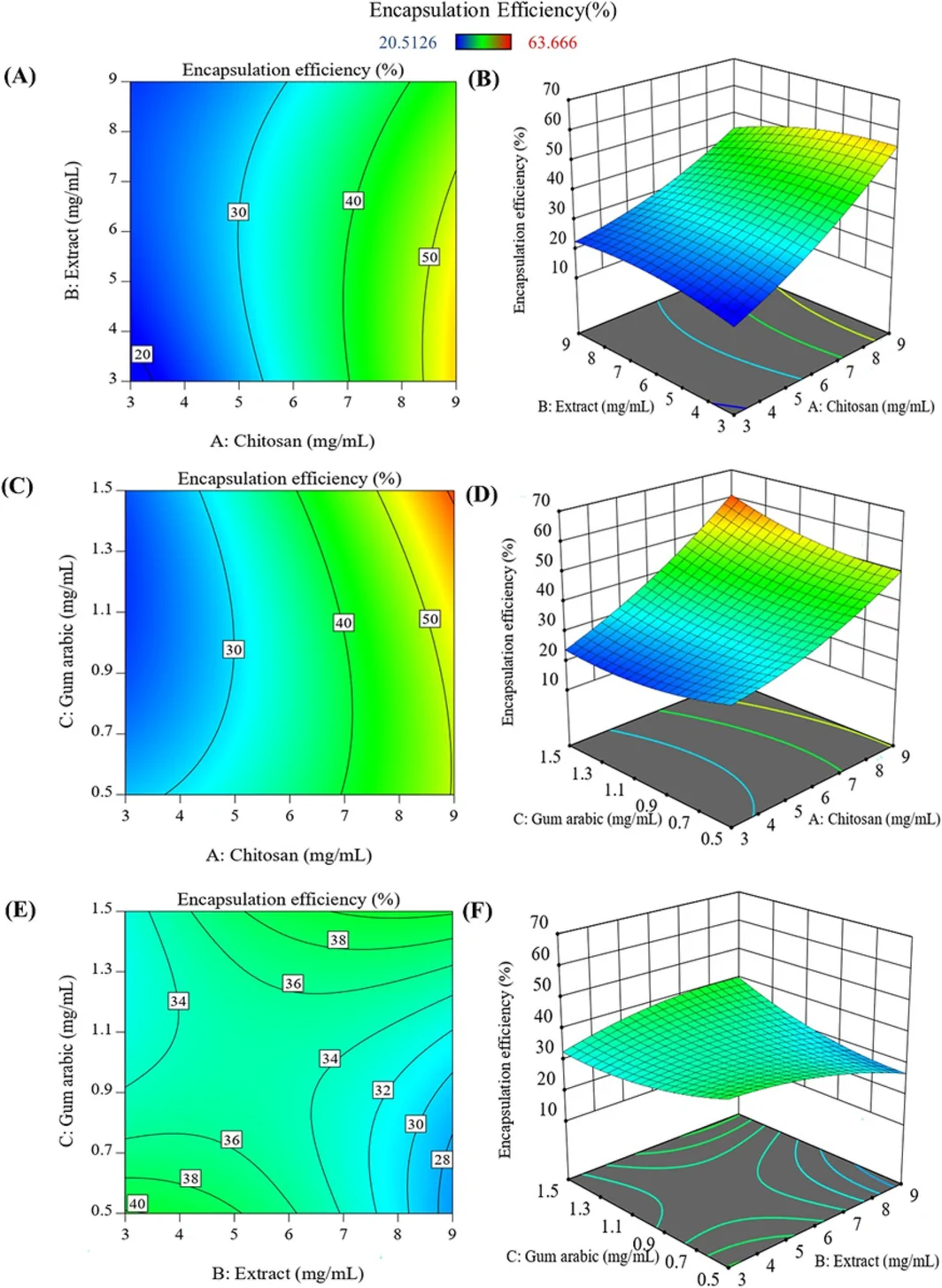
The contour and 3D surface plots for the interaction effect of different pairs of independent variables on encapsulation efficiency of nanoparticle formulations.

Figure 9 illustrates how the Chs, Ex, and GA concentrations affected the EE of the NPs, shown through 3D response surface and contour plots. Regarding the interaction effect, the contour diagram and 3D model illustrating the relationship between Ex and Chs at a medium concentration of GA (1 mg/mL) on EE are presented in Figure 9A,B. Across all Ex concentrations, an increase in Chs concentration consistently led to an improvement in EE. However, variations in Ex concentration did not significantly affect efficiency at different Chs concentrations. The lowest EE was observed at low Chs concentrations regardless of Ex concentration, whereas the highest efficiency was achieved at high Chs concentrations and Ex concentrations below the midpoint. Similarly, Rajabi et al. (2019) in the study about encapsulation of saffron bioactive by Chs–GA nanocarriers demonstrated that at stable Chs concentrations, increasing the Ex concentration resulted in relatively unchanged EE. The interaction effect between Chs and GA at a medium Ex concentration (6 mg/mL) on EE is illustrated in Figure 9C,D. Across all GA concentrations, an increase in Chs concentration led to higher EE. The impact of GA concentration on efficiency was dependent on Chs concentration. At high Chs concentrations, increasing GA improved EE, whereas at medium Chs concentrations, GA concentration exerted minimal influence. At low Chs concentrations, increasing GA concentration to moderate levels (1 mg/mL) decreased EE, but no significant changes were observed beyond this point. The highest EE was achieved at the highest concentrations of Chs (9 mg/mL) and GA (1.5 mg/mL), whereas the lowest EE occurred at the minimum Chs concentration (3 mg/mL) and intermediate GA concentrations (1 mg/mL and higher). Likewise, Kim et al. (2019) demonstrated that higher wall material concentrations enhanced EE in Chs‐GA‐NPs loaded with Quercetin due to stronger ionic gelation between Chs and GA, improving EE. The interaction effect between the Ex and GA at medium concentration of Chs (6 mg/mL) on EE is shown in Figure 9E,F. No significant interaction was observed, and EE remained within the medium range across most combinations of concentration levels.

### 3.2 Determining the Optimal Formulation

The optimal treatment was identified through the optimization module of the Design Expert software. The optimized conditions were: Chs concentration of 9 mg/mL, Ex concentration of 4.897 mg/mL, and GA concentration of 1.5 mg/mL. Under these conditions, the NPs exhibited a PS of 50 nm, ZP of 5.861 mV, EE of 60.628%, and a desirability score of 0.825, which is quite close to the theoretical maximum of 1, indicating a high desirability rate.

### 3.3 Structural and Interfacial Analysis

#### 3.3.1 Fourier Transform Infrared Spectroscopy

**FIGURE 10:**
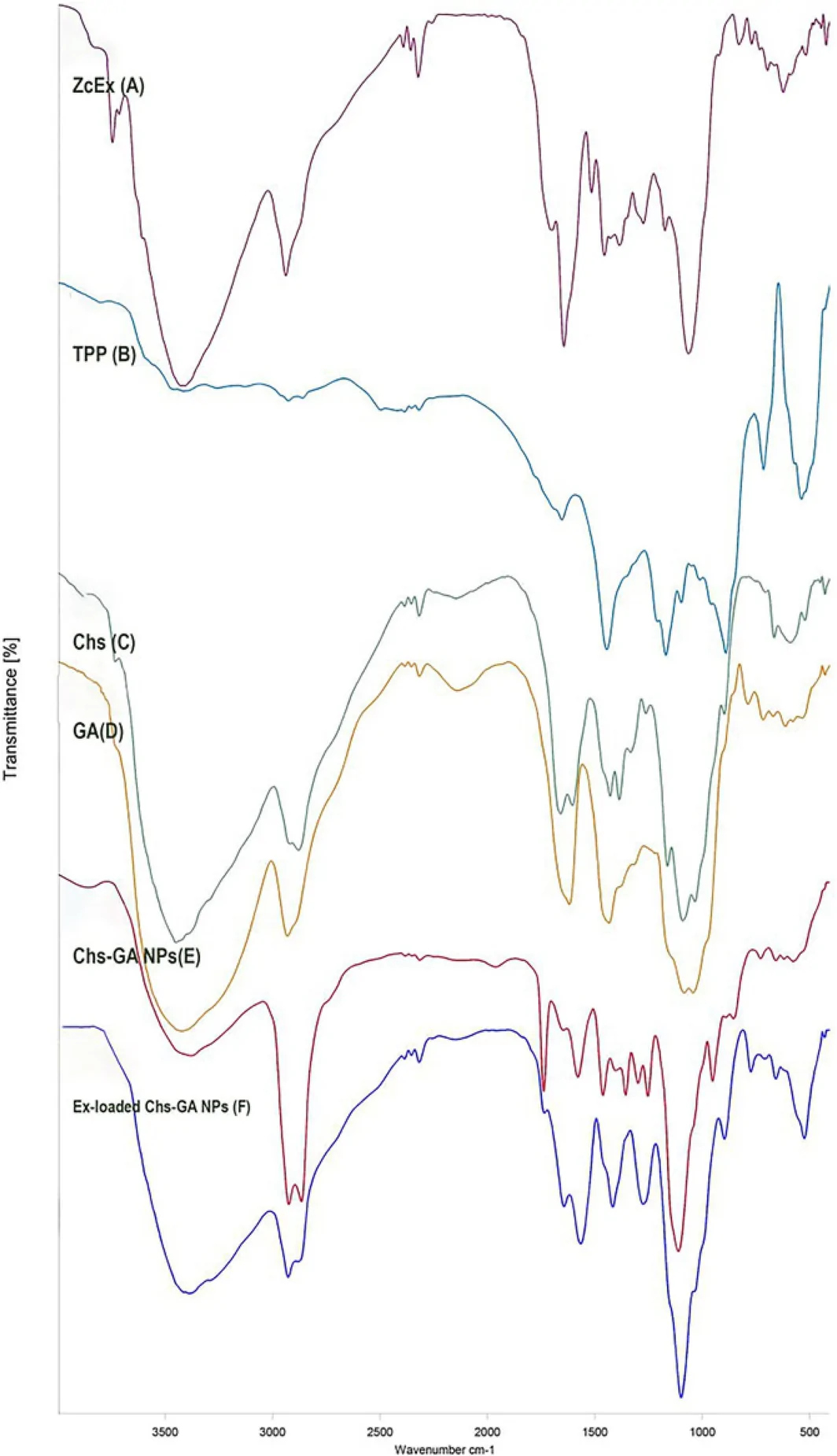
FTIR spectrum of *Ziziphora clinopodioides* L. extract (A), TPP (B), chitosan (C), gum arabic (D), chitosan and gum arabic nanoparticles (E), extract loaded chitosan and gum arabic nanoparticles (F).

FTIR spectroscopy was utilized to investigate chemical and physical interactions between the Ex component and the Chs–GA polymer matrix. The FTIR spectra obtained for individual components (Ex, TPP, Chs, and GA), the Chs–GA NPs, and the Ex‐loaded Chs–GA NPs are illustrated in Figure 10. The FTIR spectrum of the ZcEx (Figure 10A) exhibited characteristic peaks at 3394.61 cm^−1^ that indicate the presence of hydroxyl (O–H) and amino (N–H) groups, 2927.85 cm^−1^ which is attributed to the (C–H) stretching vibrations of aliphatic chains, and 1512.21 cm^−1^, which is assigned to aromatic (C=C) stretch. Yan et al. (2020) reported similar FTIR assignments for (C–H), (C=C), and (C–O) bands in Copper NPs green‐synthesized with *Nigella sativa* L. seed extract. The spectrum of TPP (Figure 10B) showed several characteristic phosphate‐related peaks. A peak at 1441.57 cm^−1^ is attributed to the (P=O) stretch and (O–H) bending. Other peaks at 886.71 cm^−1^ and 710.62 cm^−1^ correspond to (P–O–P) symmetric stretch. Chitosan (Figure 10C) displayed a peak at 3448.18 cm^−1^, corresponding to the hydroxyl (O–H) and amino (N–H) groups. The peak at 2877.61 cm^−1^ indicates (C–H) stretching vibrations. The peak at 1656.03 cm^−1^ belongs to Amide I, which is protonated to (NH_3_^+^) and disappears in Figure 10E,F. These collective features confirm the polymeric structure and characteristic functional groups of Chs. For GA (Figure 10D), a strong, broad band is visible at 3417.83 cm^−1^. This corresponds to the stretching vibrations of hydroxyl groups (O–H), which are abundant in the polysaccharide component. The absorption peak at 2928.59 cm^−1^, which is attributed to the (CH_2_) stretching of the aliphatic (C–H) groups within the sugar units. A prominent peak at 1615.94 cm^−1^ is characteristic of the GA structure. It is typically assigned to the asymmetrical stretching vibration of the carboxylate (COO^−^) groups. The strong, complex band observed at 1039.60 cm^−1^ is characteristic of polysaccharides and is assigned to the stretching vibrations of (C–O) and (C–O–C) bonds in the pyranose rings. The FTIR spectrum of Chs–GA NPs without the Ex (Figure 10E) showed significant structural changes compared to pure Chs, confirming NP formation. The disappearance of the Amide I peak (1656 cm^−1^) indicates protonation of Chs amino groups (NH_2_ → NH_3_^+^). The shift of the carboxylate peak from 1615.94 cm^−1^ in pure GA to 1576.07 cm^−1^ in the NPs demonstrates strong electrostatic interaction between the positively charged Chs (NH_3_^+^) and negatively charged GA (COO^−^), confirming ionic gelation as the primary mechanism of NP formation. A new peak at 1735.08 cm^−1^ appeared, attributed to free carboxylic acid (COOH) groups that remain unbound in the empty NPs. Notably, the characteristic phosphate peaks of TPP (1166, 886, 710 cm^−1^) were not detected, suggesting that NP formation occurred predominantly through ionic complexation rather than covalent crosslinking. A similar phenomenon was observed by Mukhopadhyay et al. (2015), who reported spectral evidence of ionic interaction in insulin NPs encapsulated by a Chs‐alginate matrix. A shift was noted in the (O–H) and (N–H) Chs stretching vibration peaks from 3427 cm^−1^ to 3469 cm^−1^ in the insulin‐loaded nanoparticle spectrum. Additionally, the significance of electrostatic interaction in this biopolymer system is supported by findings from Rajabi et al. (2019), who observed the mentioned changes in the study on encapsulation of saffron bioactive compounds by Chs–GA nanocarriers. They reported that the (N–H) bending vibration shifted from 1579 cm^−1^ to 1542 cm^−1^ and the Amide II absorbance peak shifted from 1415 cm^−1^ to 1404 cm^−1^. The FTIR spectrum of Ex‐loaded Chs–GA NPs (Figure 10F) illustrates successful encapsulation and chemical interactions between ZcEx and the polymer matrix. The characteristic phenolic (C–O) stretching peak at 1272 cm^−1^, which is prominent in the pure ZcEx (Figure 10A), appears distinctly in the loaded NPs, confirming successful incorporation of the bioactive Ex. This phenolic signature was absent in empty NPs (Figure 10E), further validating Ex loading. The ionic interaction peak shifted from 1576.07 cm^−1^ in blank NPs to 1562.51 cm^−1^ in loaded NPs, suggesting that Ex molecules interact with the Chs–GA electrostatic complex. The free carboxylic acid peak at 1735.08 cm^−1^, which was present in empty NPs, disappeared in the Ex‐loaded formulation. This indicates that the free COOH groups form hydrogen bonds with the phenolic hydroxyl groups of the Ex, facilitating encapsulation through multiple interaction mechanisms including electrostatic attraction, hydrogen bonding, and hydrophobic interactions between the aromatic components of the Ex and the polymer matrix. These peak changes are consistent with findings by Soleymanfallah et al. (2022), who reported that alterations in FTIR peak ranges and positions in grape Ex‐loaded chitosan NPs indicate precise adjustment of the glycosidic chain length during the encapsulation process.

#### 3.3.2 X‐Ray Diffraction Evaluation of Nanoparticles

**FIGURE 11:**
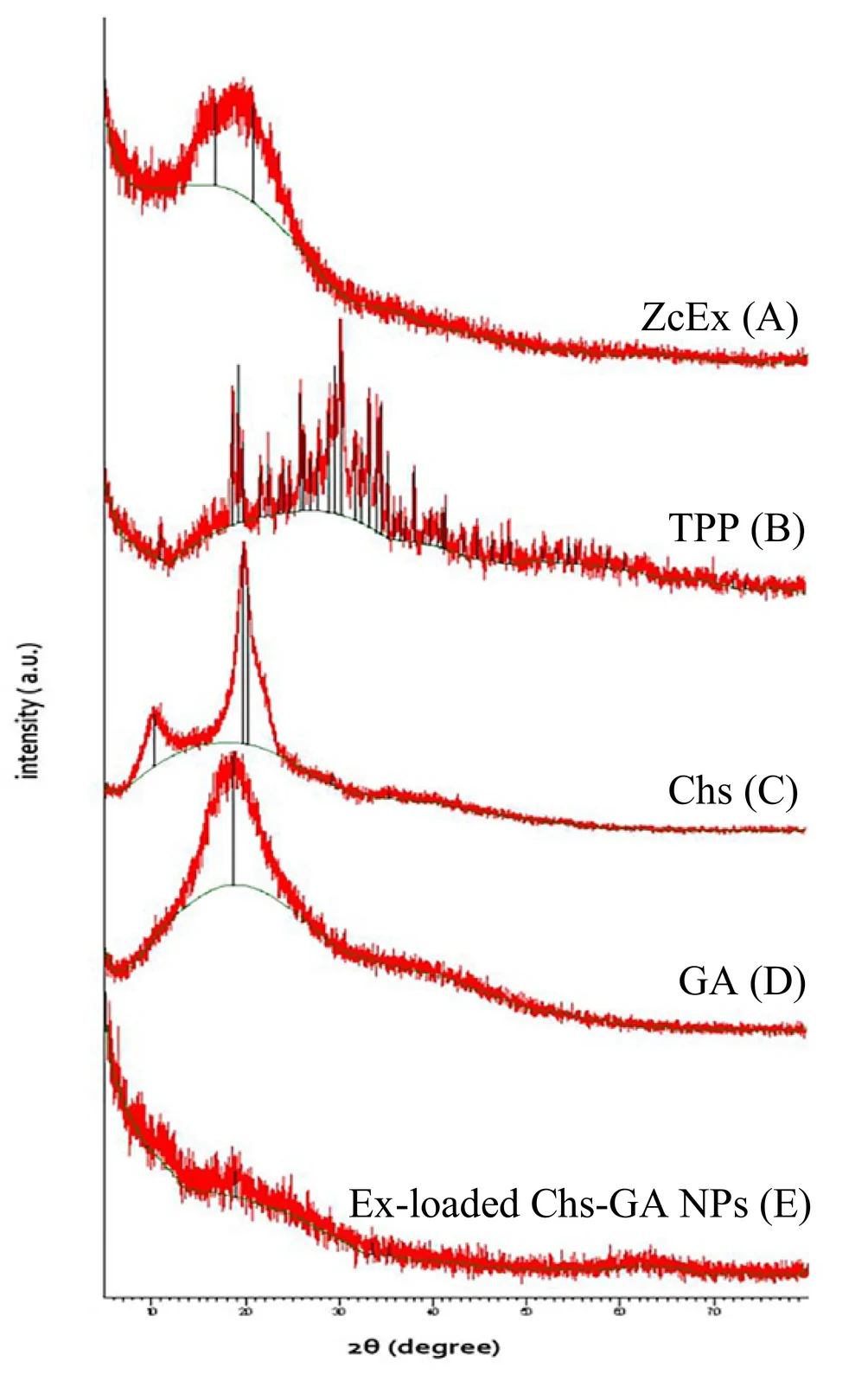
XRD spectrum of *Ziziphora clinopodioides* L. extract (A), TPP (B), chitosan (C), gum arabic (D), extract loaded chitosan and gum arabic nanoparticles (E).

The crystalline structure and phase composition of Ex, TPP, Chs, GA, blank NPs, and Ex‐loaded NPs were characterized by XRD analysis, as shown in Figure 11. The structural analysis by XRD confirms the successful formation of Ex‐loaded Chs–GA NPs by demonstrating a shift from crystalline raw materials (Figure 11A–D) to a predominantly amorphous final product. Chitosan initially showed a semi‐crystalline structure with characteristic peaks at 2*θ* = 10.51° and 2*θ* = 19.83°, and underwent a complete amorphization following complexation with amorphous GA, resulting in Ex‐loaded Chs–GA NPs with a broadly reduced crystallinity (Figure 11E). This observation is supported by the findings of Rajabi et al. (2019) on saffron bioactive encapsulation, who reported a similar disappearance of the Chs crystal peak after forming a polyelectrolyte complex with GA. Similarly, Tan et al. (2016) concluded that the crystalline structure of Chs was destroyed due to the creation of strong ionic bonding between Chs and GA during the production of curcumin‐loaded nanocapsules. Furthermore, the numerous sharp, crystalline peaks of the TPP cross‐linker (Figure 11B), most notably the intense peak at 2*θ* = 30.24°, were also completely lost in the final NP structures, confirming the extensive ionic cross‐linking between the biopolymers and TPP. This complete disappearance of TPP's crystalline signature is consistent with findings by Haider et al. (2017), who reported that electrostatic interaction with TPP resulted in reduction of peak intensity in Chs NPs. Ex‐loaded Chs–GA NPs exhibited loss of the original characteristic peaks from both the Chs and Ex components. As shown in Figure 11E, the absence of sharp peaks corresponding to original Ex, coupled with the observation of a broad, amorphous hump at 2*θ* = 18.68° indicates that the Ex molecules are no longer present in a bulk crystalline state. This successful entrapment and transformation into an amorphous structure is a key indicator of effective encapsulation, correlating with enhanced solubility and bioavailability. These findings align with observations reported by Soleymanfallah et al. (2022), who noted that the distinctive crystalline peaks of grape Ex (2*θ* = 21.57°) and Chs NPs (2*θ* = 21.57°) merged upon encapsulation, resulting in a single broad peak (2*θ* = 23°). This shift occurs due to the molecular dispersion of the bioactive compounds of Ex within the polymer matrix of Chs, preventing the formation of ordered crystalline structures.

## 4 Conclusion

This study optimized the formulation of Chs‐GA‐NPs loaded with ZcEx, including Chs, ZcEx, and GA concentrations, using RSM. The results demonstrated the statistically significant effects of varying concentrations of ZcEx, Chs, and GA on critical response variables, including PS, ZP, and EE. Among the factors investigated, chitosan had the highest effect on the studied responses.

A negative correlation was observed between Ex concentration and EE. Conversely, GA acted as a stabilizing agent that positively influenced the EE as its concentration increased. However, increasing Ex concentration led to an increase in PS and ZP, and ZP was also enhanced by increasing Chs concentrations. Optimal conditions yielded NPs with a PS of 50 nm, a ZP of 5.86 mV, and an EE of 60.63. The FTIR and XRD analyses confirmed proper encapsulation of the extract. The findings emphasize the potential of Chs and GA as biocompatible and effective carriers for encapsulating plant extracts. These results contribute to the current understanding of NP delivery systems and their application in improving the potential efficacy of natural compounds. However, the highest extract concentration investigated in this study was limited to 9 mg/mL. Therefore, it is highly recommended that future research investigate a wider concentration range of the extract, considering our established 9 mg/mL as an intermediate level, to achieve further formulation optimization. Moreover, future studies should focus on evaluating the biological activity and stability of these NPs in real world applications, particularly in food preservation.

## Author Contributions

**Anita Ehterami:** writing – original draft, investigation, formal analysis, data curation, software. **Hassan Gandomi:** conceptualization, validation, writing – review and editing, supervision, project administration, visualization. **Mohammad Kazem Koohi:** supervision. **Negin Noori:** methodology. **Masoud Kazeminia:** investigation, formal analysis, data curation, software, resources.

## Conflicts of Interest

The authors declare no conflicts of interest.

## Data Availability

The datasets generated and analyzed during the current study are not publicly accessible, but can be obtained from the corresponding author upon reasonable request.
